# Emerge of NDM-1-Producing Multidrug-Resistant *Pseudomonas aeruginosa* and Co-Harboring of Carbapenemase Genes in South of Iran

**Published:** 2020-05

**Authors:** Ahmad FARAJZADEH SHEIKH, Mojtaba SHAHIN, Leili SHOKOOHIZADEH, Fahimeh GHANBARI, Hamid SOLGI, Fereshteh SHAHCHERAGHI

**Affiliations:** 1. Department of Microbiology, School of Medicine, Ahvaz Jundishapur University of Medical Sciences, Ahvaz, Iran; 2. Health Research Institute, Infectious and Tropical Diseases Research Center, Ahvaz Jundishapur University of Medical Sciences, Ahvaz, Iran; 3. Department of Medical Laboratory Sciences, Faculty of Medical Sciences, Arak Branch, Islamic Azad University, Arak, Iran; 4. Department of Microbiology, Faculty of Medicine, Hamadan University of Medical Sciences, Hamedan, Iran; 5. Student Research Committee, Shahid Sadoughi University of Medical Sciences, Yazd, Iran; 6. Department of Bacteriology, Microbiology Research Center, Pasteur Institute of Iran, Tehran, Iran

**Keywords:** *Pseudomonas aeruginosa*, New Delhi metallo-β-lactamase (*bla_NDM-1_)*, Modified hodge test (MHT), Double-disk potentiation tests (DDPT), Double disk synergy test (DDST)

## Abstract

**Background:**

New Delhi metallo**-**beta**-**lactamase*-*1 (NDM-1) is one of the most important emerging antibiotic resistance. Co-harboring three or four carbapenemases is rare and only a few reports exist in the literature. We described the characteristics of the large epidemic outbreaks and reports co-producing *bla_NDM-1_* with the other carbapenemase genes in *P. aeruginosa* isolates.

**Methods:**

This present cross-sectional research was conducted on 369 *P. aeruginosa* isolates obtained from burn and general hospitals within years 2013 to 2016. Beta-lactamase classes A, B and D genes were identified by PCR method. Modified hodge test (MHT), double-disk potentiation tests (DDPT) and double disk synergy test (DDST) were performed for detection carbapenemase and metallo beta-lactamase (MBL) production of *bla_NDM-1_* positive *P. aeruginos* isolates.

**Results:**

From 236 carbapenem-resistant *P. aeruginosa* (CRPA), 116 isolates have had MBL genes and twenty-nine isolates were found positive for *bla_NDM-1_*. In CRPA isolates, *bla_IMP-1_*, *bla_VIM-2_* and *bla_OXA-10_* were identified in 27.5%, 21.1% and 32.2% of isolates respectively, while co-producing *bla_NDM-1_*, *bla_IMP-1_*, *bla_OXA-10_*, co-producing *bla_NDM-1_*, *bla_VIM-2_*, *bla_OXA-10_* and co-producing *bla_IMP-1_*, *bla_VIM-2_* were determined in 11 (4.6%), 8 (3.4%) and 27 (11.4%) of isolates respectively.

**Conclusion:**

The finding of this co-existence of multiple carbapenemase resistance genes is threating for public health. Dipicolinic acid is a superior MBL inhibitor in DDPT antique than EDTA in DDST method for the detection of MBL-*bla_NDM-1_* producing *P. aeruginosa*

## Introduction

*Pseudomonas aeruginosa* is major agents of hospital-acquired pathogens (1). Carbapenemases indicate the most versatile family of beta-lactamase, with a wide spectrum inimitable by other beta-lactam hydrolyzing enzymes (2).

Carbapenems are the last-line treatment of multi-drug-resistant *P. aeruginosa* (MDRP) infections (1, 3). Because of the fact that carbapenems are a last resort treatment choice for infections caused by MDRP isolates, the presence of carbapenem-resistant strains is becoming a main public health challenge (2, 3). Among plasmid-mediated, extended-spectrum beta-lactamases (ESBLs) are commonly known to hydrolyze cephalosporins and metallo beta**-**lactamase**s** (MBLs) can hydrolyze carbapenems.

Resistance to carbapenems can be related to producing carbapenemase enzymes such as serine carbapenemases (containing KPC and GES enzymes) and MBLs **(**metallo**-**beta**-**lactamases**)** such as imipenemase (IMP), Verona integrin-encoded metallo-β-lactamase (VIM) and New Delhi metallo-β-lactamase (NDM), enzymes and oxacillinases (such as OXA enzymes) (2, 4, 5).

MBLs such as *bla_VIM_* and *bla_IMP_* are the most clinically important classes of beta-lactamases; but the lately discovered transmissible New Delhi metallo beta**-**lactamase*-*1 (NDM-1) is becoming the most menacing in carbapenemase genes (2, 6). In addition, most *bla_NDM-1_* strains are resistant to a wide-ranging of other antibiotic groups and transport numerous additional resistance genes for example to aminoglycosides, sulfonamides, macrolides and fluoroquinolones (7).

The detection of this co-harboring of multiple carbapenem resistance genes (Simultaneous attendance of both MBL and non-MBL genes) in clinical isolates from supremacy of carbapenems are considered as the last line resort of option for most of the dangerous infections caused by *P. aeruginosa*, but due to the prevalence of carbapenem-resistant *P. aeruginosa* (CRPA) isolates these lifesaving antibiotics were compromised in treating the patients with serious sickness (8). The aims study were to identify the carbapenemase classes A, B and D and ESBL determinants among CRPA isolates in burn and non-burn patients. Moreover, identification of *bla_NDM-1_* by three phenotypic methods (include DDPT, DDST and MHT) and comparing with PCR method was evaluated.

## Materials and Methods

### Bacterial isolation and identification

During the period from Oct 2013 to Jul 2016, 369 non-duplicate isolates were collected in burns (102 isolates from burn wounds**)** and general hospitals (267 from various hospital wards**).** These isolates were collected from teaching hospitals’ microbiology laboratories in Ahvaz, Isfahan and Tehran cities from Iran. The isolation and identification of *P. aeruginosa* were done by the conventional methods and proved by PCR amplification with specific primers for *P. aeruginosa* gyrB gene with product size 221bp (9).

### Antimicrobial susceptibility testing

The antibiotic susceptibility of all the isolates was tested by employing the Kirby-Bauer’s technique as suggested by the CLSI (10). The eleven antibiotic disks used include: imipenem (10 μg), meropenem (10 μg), ertapenem (10 μg), ciprofloxacin (5 μg), ceftazidime (30 μg), cefepime (30 μg), cefotaxime (30 μg), amikacin (30 μg), gentamicin (10 μg), piperacillin/tazobactam (100/10 μg), aztreonam (30 μg) (Mast Group Ltd, UK). Isolates with resistance against a minimum of three groups of antibacterial agents were considered as MDR (11). To detect ESBL phenotype combined disk method using disks of ceftazidime (30 mg) with (10 mg) and without clavulanic acid (Mast Group Ltd, UK) was applied to all positively screened isolates by modified hodge test (MHT) (11). A growth in the area diameter of ≥5 mm around ceftazidime disc with and without clavulanic acid was expected to be a positive result for ESBL production (12, 13). The MHT was performed for all isolates as recommended by CLSI (10). The E test (imipenem 0.002–32μg/mL) (Liofilchem, Roseto degli Abruzzi, Italy) was applied (*according to the manufacturer’s* instructions) to all positively screened isolates by PCR test for *bla_NDM_* gene, to determine minimum inhibitory concentrations (MICs).

### Phenotypic detection of MBLs

The double-disk potentiation tests (DDPT) and double disk synergy test (DDST) was performed for all *bla_NDM-1_* positive (14, 15) for phenotypic detection of *bla_NDM-1_* producing isolates*. The bacterial suspension with turbidity equivalent to 0.5 McFarland standard was prepared and cultured on MH agar.* Two *imipenem and imipenem*-EDTA disks and meropenem+Dipicolinic acid (Liofilchem, Roseto degli Abruzzi, Italy) were placed on the surface of the agar at a distance of 4 cm from each other. After 18–24 h of incubation at 35–37 °C, the inhibition zone of imipenem disks with imipenem alone and disks with imipenem plus 750 μg of EDTA were measured. An increase of 7 mm or more in the zone diameter for imipenem-EDTA disk in comparison with imipenem disk alone was considered as a MBLs producing isolate. Moreover, DDPT was interpreted as positive even if a small potentiation inhibition zone was present (14, 15).

### PCR amplification of resistance genes

**Fig. 1:**
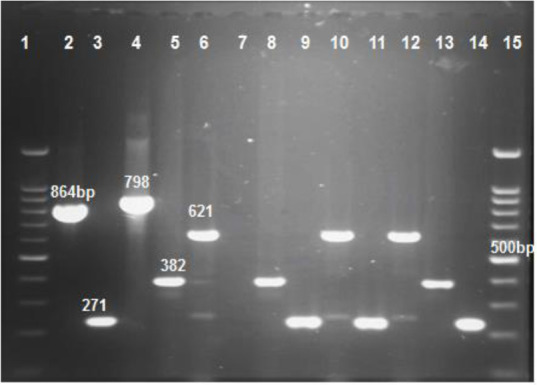
Gel electrophoresis of multiplex PCR products following amplification with specific primers. Line 1 and 15 ladder, line2, 3, 4, 5 and 6 positive control *bla_KPC_*, *bla_IMP_, bla_GES_*, *bla_VIM_* and *bla_NDM_* (864, 271, 798, 382 and 621 bp respectively), line 7 deionized water as control negative, line 8–14 samples. All positive controls were provided by the Pasteur institute Iran

DNA of strains was extracted by the DNA extraction set (Sinaclon, Iran) based on *the* guidelines *of the manufacturer*. The specific primers were used for different types of carbapenemase (*bla_NDM_*, *bla_IMP_*, *bla_VIM_*, *bla_KPC_*, *bla*_GES_, *bla_SPM_* and *bla_OXA-10_*). In this study, pentaplex PCR was used for the rapid detection of MBL genes in CRPA isolates. The pentaplex PCR was optimized successfully to identify the MBL genes. Stepwise optimization of annealing temperature, primer concentration, MgCl_2_, dNTP and Taq polymerase was performed. The pentaplex PCR gave the excellent results when 5 μL of 10X reaction buffer, 2 μL of 50 mM MgCl2, 1.5 μL of 2.5 mM dNTPs, 0.25 μL of each 10 pmol/μL primer, 0.5 μL Taq polymerase 5 U/μL, 37 μL distilled water and 55 °C annealing temperature were used (Fig. 1). The amplification reactions were carried out in a thermal cycler (Eppendorf AG, Germany), with an initial denaturation 4 min at 94 °C followed by 30 cycles of denaturation 60 sec at 94 °C, annealing 56 °C for *bla_OXA-10_*, 59 °C for *bla_SPM_* and 55 °C for pentaplex PCR and extension 60 sec at the temperature of 72 °C, with a single final extension of 7 min at 72 °C. The size of PCR products is determined by comparison with a DNA ladder (Sinaclon, Iran) on 1.5% agarose gels stained with ethidium bromide. Sequencing of the amplicons was performed by the Bioneer Company (Bioneer, Daejeon, South Korea). The nucleotide sequences were analyzed using blast in NCBI.

### Ethics approval

This study was approved by the Medical Ethics Committee of Ahvaz Jundishapur University of Medical Sciences in Iran approved the study (permit number IR.AJUMS.REC.1395.227).

## Results

Totally, of 369 confirmed *P. aeruginosa* isolates, 219 (59.3%) isolates were obtained from male and 150 (40.7%) isolates from female subjects. The majority 113 (30.6%) of isolates were obtained from punch/wound followed by 22.7% (84/369) from tracheal tube and 21.4% (79/369) isolates from urine samples. Seventy-four percent of all isolates were MDR (84% burn isolates and 67% from various hospital wards). Among all isolates, 267 (72.3%) were carbapenem-resistant, meanwhile, the highest sensitivity was against to piperacillin-tazobactam 157 (42.5%). The full results of antibiotic resistance pattern of *P. aeruginosa* isolates shown in Table 1. MHT results showed that 236/369 (63.9%) isolates were positive as CRPA. Among CRPA isolates, high-level resistance to imipenem, meropenem and cefotaxime was observed. The comparison of antibiotic resistance of the CRPA in burn and non-burns isolates are shown in Table 2. Of 236 CRPA, 116 isolates (21 burn isolates and 95 isolates from various hospital wards) were MBL producing isolates, moreover, 105 (90.5%) were MDR isolates. In particular, this collection was included non-duplicate characterized *bla_VIM_*, *bla*_IMP_ and *bla*_NDM-1._

**Table 1:** Antimicrobial susceptibility results of the all *Pseudomonas aeruginosa* isolates

***Antimicrobial agent***	***The number of P. aeruginosa***	***Number of Sensitive (%)***	***Number of Intermediate (%)***	***Number of Resistant (%)***
Imipenem	369	118(32)	22(5.9)	229(62.1)
Meropenem	369	118(32)	12 (3.2)	239(64.8)
Ertapenem	369	92(25)	10 (2.7)	267(72.3)
Piperacillin-	369	140(38)	72(19.5)	157(42.5)
Tazobactam				
Cfepime	369	130(35.3)	23(6.2)	216(58.5)
Amikacin	369	169(45.8)	20(5.4)	180(48.8)
Ciprofloxacin	369	120(32.5)	20(5.4)	229(62.1)
Gentamicin	369	134(36.3)	0	235(63.7%)
Ceftazidime	369	150(40.7)	16(4.3)	203(55)
Cefotaxime	369	25(6.8)	65(17.6)	279(75.6)
Azteronam	369	114(30.9)	121(32.8)	134(36.3)

**Table 2:** Antimicrobial susceptibility results of the CRPA in burns and non-burns isolates

***Antimicrobial agent***	***The number of CRPA isolates***		***Sensitive (%)***		***Intermediate (%)***		***Resistant (%)***	
	*Burn patients*	*Non-burn patients*	*Burn patients*	*Non-burn patients*	*Burn patients*	*Non-burn patients*	*Burn patients*	*Non-burn patients*
Imipenem	78	158	5(6.4)	7(4.4)	4(5.2)	6(3.8)	69(88.4)	145(91.8)
Meropenem	78	158	5(6.4)	6(3.8)	3(3.8)	8(5.1)	70(89.8)	144(91.1)
Ertapenem	78	158	2(2.6)	16(10.1)	2(2.6)	8(5.1)	74(94.8)	134(84.8)
Piperacillin-tazobactam	78	158	1(1.3)	46(29.1)	6(7.7)	50(31.6)	71(91)	62(39.2)
Cefepime	78	158	1(1.3)	26(16.4)	7(8.9)	11(7)	70(89.8)	121(76.6)
Amikacin	78	158	3(3.8)	64(40.5)	3(3.8)	12(7.6)	72(92.4)	82(51.9)
Ciprofloxacin	78	158	2(2.6)	23(14.5)	3(3.8)	8(5.1)	73(93.6)	127(80.4)
Gentamicin	78	158	4(5.2)	31(19.6)	0	0	74(94.8)	127(80.4)
Ceftazidime	78	158	21(26.9)	23(14.5)	7(8.9)	4(2.5)	50(64.2)	131(83)
Cefotaxime	78	158	2(2.6)	1(0.6)	2(2.6)	20(12.7)	74(94.8)	137(86.7)
Azteronam	78	158	8(10.2)	39(24.7)	32(41.1)	54(34.2)	38(48.7)	65(41.1)
Total	236(63.9)							
	CRPA isolates							

The presence of *bla*_IMP_ and *bla*_VIM_ gene were detected in 21.6% (51/116) and 28.8% (68 isolates) of MBL producing isolates, respectively. The full results of antibiotic resistance pattern of *bla_IMP_* and *bla_VIM_* positive isolates in burns and non-burns isolates showed in Tables 3 and 4.

**Table 3:** Antimicrobial susceptibility results of VIM positive in burns and non-burns isolates

***Antimicrobial agents***	***The number of CRPA carrying VIM gene No. (%)***		***Sensitive No. (%)***		***Intermediate No. (%)***		***Resistant No. (%)***	

	Burn patients	Non-burn patients	Burn patients (%)	Non-burn patients (%)	Burn patients (%)	Non-burn patients (%)	Burn patients (%)	Non-burn patients (%)
Imipenem	18(17.6)	33(12.4)	1(5.5)	0	3(16.7)	2(6.1)	14(77.7)	31(93.9)
Meropenem	18(17.6)	33(12.4)	1(5.5)	1(3)	0	0	17(94.5)	32(97)
Ertapenem	18(17.6)	33(12.4)	1(5.5)	2(6.1)	0	1(3)	17(94.5)	30(90.9)
Piperacillin-tazobactam	18(17.6)	33(12.4)	1(5.5)	16(48.5	2(11.1 )	12(36.4)	15(83.4)	5(15.1)
Cefepime	18(17.6)	33(12.4)	0	9(27.3)	1(5.5)	1(3)	17(94.5)	23(69.7)
Amikacin	18(17.6)	33(12.4)	2(11.1)	25(75.8)	1(5.5)	3(9.1)	15(83.4)	5(15.1)
Colistin	18(17.6)	33(12.4)	18(100)	32(97)	0	0	0	1(3)
Ciprofloxacin	18(17.6)	33(12.4)	0	7(21.2)	1 (5.5%)	2(6.1)	17(94.5)	24(72.7)
Gentamicin	18(17.6)	33(12.4)	0	9(27.3)	0	0	18(100)	23(82.3)
Ceftazidime	18(17.6)	33(12.4)	1(5.5)	7(21.2)	1(5.5)	1(3)	16(89)	25(75.8)
Cefotaxime	18(17.6)	33(12.4)	1(5.5)	0	1(5.%)	5(15.1)	16(89)	28(84.9)
Azteronam	18(17.6)	33(12.4)	3(16.7)	11(33.3)	2(11.1)	13(39.4)	13(72.2)	9(27.3)
Total		51 VIM isolates						

**Table 4:** Antimicrobial susceptibility results of IMP positive in burns and non-burns isolates

***Antimicrobial agents***	***The number of CRPA carrying IMP gene No. (%)***		***Number of Sensitive No. (%)***		***Number of Intermediate No. (%)***		***Number of*** ***Resistant No. (%)***	

	Burn patients	Non-burn patients	Burn patients (%)	Non-burn patients (%)	Burn patients (%)	Non-burn patients (%)	Burn patients (%)	Non-burn patients (%)
Imipenem	21(20.6)	47(17.6)	0	1(2.1)	0	1(2.1)	21(100)	45(95.8)
Meropenem	21(20.6)	47(17.6)	0	1(2.1)	0	0	21(100)	46(97.9)
Ertapenem	21(20.6)	47(17.6)	0	2(4.2)	1(4.8)	0	20(95.2)	45(95.8)
Piperacillin-	21(20.6)	47(17.6)	1(4.8)	17(26.5)	2(9.5)	26 (41.2)	18(85.7)	4(32.3)
Tazobactam								
Cefepime	21(20.6)	47(17.6)	2(9.5)	9(19.2)	1(4.8)	1(2.1)	18(85.7)	37(78.7)
Amikacin	21(20.6)	47(17.6)	1(4.8)	23(48.95)	1(4.8)	1(2.1)	19(90.4)	23(48.95)
Colistin	21(20.6)	47(17.6)	21(100)	45 (95.8)	0	0	0	2(4.2)
Ciprofloxacin	21(20.6)	47(17.6)	2(9.5)	5(10.6)	1(4.8)	1(2.1)	18(85.7)	41(87.3)
Gentamicin	21(20.6)	47(17.6)	1(4.8)	6(12.7)	0	0	20(95.2)	41(87.3)
Ceftazidime	21(20.6)	47(17.6)	2(9.5)	4(8.5)	1(4.8)	2(4.2)	18(85.7)	41(87.3)
Cefotaxime	21(20.6)	47(17.6)	0	0	0	3(6.4)	21(100)	44(93.6)
Azteronam	21(20.6)	47(17.6)	4(19)	7(14.9)	5(23.8)	18 (38.3)	12(57.2)	22(46.8)
Total	68 IMP isolates							

Twenty-four isolates from Ahvaz, 4 isolates from Isfahan and one isolate from Tehran in the collection was found carrying bla_**NDM**-1_ and confirmed by sequencing. The prevalence of ESBLs in MBL isolates was 11.2% (13/116) that 3 of them were *bla_NDM-1_* isolates. Nineteen of *bla_NDM-1_* isolates were co- harboring of two genes (*bla_VIM-__2_***/***bla_OXA-10_* and *bla_IMP-1_***/***bla_OXA-10_*). Moreover, two *bla_NDM-1_* isolates were co-harboring of three genes (*bla_VIM-2_*, *bla_IMP-1_* and *bla_OXA-10_*). Moreover, 86.2% (25) *bla*_NDM_*_-_*_1_ positive isolates contained *bla_oxa-10_*, simultaneously. Furthermore, *bla_KPC_*, *bla_GES_* and *bla_SPM_* genes not found in none of the *bla*_NDM_*_-_*_1_ positive isolates. Unexpectedly, the results of DDST and DDPT revealed that 15(51.8%) and 26 (89.7%) of *bla_NDM-1_* positive isolates were MBL producing isolates, respectively.

## Discussion

Previously, only producers of the MBLs *bla_VIM_* and *bla_IMP_* had been detected*. bla_NDM-1_* producing strains are surely threatening: firstly, *bla_NDM-1_* encoding plasmids co-carriage multiple resistance determinants, they are commonly accounted as MDR isolates. Secondly, *bla_NDM-1_* positive isolates have a potential for extent through the transfer of the plasmid *bla_NDM_* gene (16). As explained previously, there are rare published reports of *bla_NDM-1_* co-existence of multiple carbapenem resistance genes.

Infections with *bla_NDM-1_* producing isolates in non-endemic regions such as Europe and North America are often linked to visit and be hospitalized in endemic regions such as Indian subcontinent (17). The first report of *bla_NDM-1_* positive in *P. aeruginosa* came from Serbia (18). *bla_NDM-1_* producing *P. aeruginosa* is extremely rare (19). To date there are no reports of co-harboring occurrence *bla_NDM-1_* in *P. aeruginosa* isolates in Iran.

Nevertheless, *P. aeruginosa* isolates producing three carbapenemase genes is rare and has been reported in Brazil *(bla*_SPM-1_*, bla*_KPC-2_* and bla*_VIM-2_*) (20)*, Denmark (*bla_NDM-1_*, *bla_VIM_*_-2_, *bla_IMP_*_-1_) (8), Bangladesh (*bla_NDM-1_*, *bla_VIM-2_*, *bla_IMP-1_*) (21) and Turkey (*bla_VIM-1_*, *bla_VIM-2_*, and *bla_GES-5_*) (22). Although these cases are scarce and sporadic, information of its occurrence is vital because NDM-positive *P. aeruginosa* is an organism with potent colonization ability in the hospital for long periods (23). To best of our knowledge, we report the first report of *P. aeruginosa* isolates producing four carbapenemases *co-existence bla_NDM-1_*, *bla_VIM-2_*, *bla*_IMP-1_ and *bla_OXA-10_* from Iran*.* The acquisition of MBL-carbapenemase *bla_NDM-1_*, *bla_VIM_*, *bla_IMP_* and *bla_SPM_* led to emergence of MDR or XDR *P. aeruginosa (16)*.

In the present study, imipenem resistance in burn and non-burn patients was 83.2% and 57.5% respectively. Imipenem was the ninth and fourth effective drugs in burn and non-burn isolates respectively, while in other researches particularly on burned patients in Iran, it was the most effective antipseudomonal antibiotic (24) in 10.8% of 415 isolates In burn patients, ceftazidime (with 26.9% sensitivity (and ertapenem, gentamicin and cefotaxime (with 94.8% resistance (and in non-burn patients amikacin (with 40.5% sensitivity) and imipenem (with 91.8%) resistance were the most and least effective antipseudomonal antibiotics. Even though, amikacin is the most effective antibiotic for infection of CRPA isolates, and also is a good drug for the treatment of non-burn isolates in CRPA isolates, but interestingly, we found that amikacin was a poorly antibiotic for burn infections due to CRPA isolates, the rate of resistance to this antibiotic was 92.4% which is relatively high in burn isolate. Similar to current study, another study among burned patients, reported 97.5% of *P. aeruginosa* isolates were resistant to imipenem and 90% of isolates resistant to amikacin (25). In Isfahan, surveyed 106 *P. aeruginosa* was isolated and 62 (58.5%) of isolates were imipenem resistance also MBL detected in 26| (42%) of them (26). In the current study, 21.6% and 28.8% of MBL producing strains, carried *bla_IMP_* and *bla_VIM_*, respectively. This rate is slightly higher than the result reported in previous studies, which can be a serious concern that may be because of a general increase in the extent of attainment of MBL genes among *P. aeruginosa*. This genes are found to be located on the class I integron and can hence quickly transfer among *P. aeruginosa* strains (27, 28). Compared to present study, lower resistant to imipenem (n=26, 25.2%), which 19 (73.0%) of them produce MBL, 6 (31.5%) samples had *bla_VIM_* gene and 2 (10.5%) had *bla_IMP_* gene. Lower percentage of IMP expression (10.5%) than our study has been also reported (29). One general concept has been evidenced that the quick appearance and dissemination of carbapenemase-producing strains is mostly due to the acquisition of *bla_NDM_* and *bla_VIM-2_* (7, 28). Antimicrobial susceptibility results of VIM and IMP positive isolates in burns and non-burns isolates indicated that high resistance to antibiotics. The corporation of other resistance determinants along with *bla*_VIM_ confers the phenotype to become resistant to most of the accessible antibiotics (28). Aminoglycosides resistance genes on the similar gene cassette along with *bla_VIM_*_-2_, therefore making the phenotype resistance to gentamicin and amikacin as well (30). Recognition of MBL-producing isolates can be effective for correct treatment of patients especially in burned patients (2). The mortality rate of patients infected with MBL-producing *P. aeruginosa* was higher (51.2%) than mortality caused by non-MBL-producing strains (32.1%) (31). Aztreonam is not appreciably hydrolyzed by NDM enzymes. Aztreonam was more effective than the carbapenems (31), but our study showed that 62% of these isolates were resistant to aztreonam. This occurrence of *bla_OXA-10_* was inside the range by Golshani (64%), Mirsalehian (74%), but more than other areas; however, *bla_OXA-10_* is prevalent in *P. aeruginosa* (24, 32).

Several phenotypic methods to detect MBL production have been developed, comprising the MHT, DDST, DDPT and E-test (15, 33). The MHT is the only CLSI recommended carbapenemase-screening method detected the weak carbapenemase activity enzyme. However, PCR is specific for detection of *bla_NDM_*. The reports have shown a poor sensitivity of DDST and MHT phenotypic technique for detection *bla_NDM_*, furthermore, due to its high false negative results, evaluating the performance of the MBL are needed (15, 33, 34). In the present study, 51.8% and 89.7% *bla_NDM-1_* isolates were positive in DDST and DDPT methods. There is a need for a more thorough evaluation of *bla_NDM_*.in *P. aeruginosa* (35,36).

## Conclusion

These findings imply the importance of *bla_NDM-1_* screening in Iran, which are being reported as potential regions of *bla_NDM-1_* endemicity. The emergence of an acutely drug-resistant strain carrying multiple carbapenemase genes is threating global health. Dipicolinic acid is a superior MBL inhibitor in DDPT than EDTA in DDST method for the detection of MBL-*bla_NDM-1_* producing *P.aeruginosa*. More research is needed to detect the *bla_NDM-1_* source.

## Ethical considerations

Ethical issues (Including plagiarism, informed consent, misconduct, data fabrication and/or falsification, double publication and/or submission, redundancy, etc.) have been completely observed by the authors.

## References

[1] Faghri J Nouri S Jalalifar S Zalipoor M Halaji M (2018). Investigation of antimicrobial susceptibility, class I and II integrons among Pseudomonas aeruginosa isolates from hospitalized patients in Isfahan, Iran. BMC Res Notes, 11(1):806. 30419962 10.1186/s13104-018-3901-9 PMC6233361

[2] Queenan AM Bush K (2007). Carbapenemases: the versatile beta-lactamases. Clin Microbiol Rev, 20(3):440–58. 17630334 10.1128/CMR.00001-07 PMC1932750

[3] Jamal WY Albert MJ Rotimi VO (2016). High Prevalence of New Delhi Metallo-beta-Lactamase-1 (NDM-1) Producers among Carbapenem-Resistant Enterobacteriaceae in Kuwait. PLoS One, 11(3):e0152638. 27031521 10.1371/journal.pone.0152638 PMC4816385

[4] Farajzadeh Sheikh A Rostami S Jolodar A (2014). Detection of Metallo-Beta Lactamases Among Carbapenem-Resistant *Pseudomonas aeruginosa*. Jundishapur J Microbiol, 7(11):e12289. 25774271 10.5812/jjm.12289 PMC4332233

[5] Rezaei A Fazeli H Moghadampour M Halaji M Faghri J (2018). Determination of antibiotic resistance pattern and prevalence of OXA-type carbapenemases among Acinetobacter baumannii clinical isolates from inpatients in Isfahan, central Iran. Infez Med, 26(1):61–66. 29525799

[6] Jovcic B Lepsanovic Z Begovic J (2014). Two copies of blaNDM-1 gene are present in NDM-1 producing Pseudomonas aeruginosa isolates from Serbia. Antonie Van Leeuwenhoek, 105(3):613–8. 24343100 10.1007/s10482-013-0094-z

[7] Paul D Dhar D Maurya AP (2016). Occurrence of co-existing bla VIM-2 and bla NDM-1 in clinical isolates of *Pseudomonas aeruginosa* from India. Ann Clin Microbiol Antimicrob, 15:31. 27154587 10.1186/s12941-016-0146-0 PMC4859973

[8] Wang M Borris L Aarestrup FM (2015). Identification of a Pseudomonas aeruginosa co-producing NDM-1, VIM-5 and VIM-6 metallo-beta-lactamases in Denmark using whole-genome sequencing. Int J Antimicrob Agents, 45(3):324–5. 25542060 10.1016/j.ijantimicag.2014.11.004

[9] Lavenir R Jocktane D Laurent F (2007). Improved reliability of *Pseudomonas aeruginosa* PCR detection by the use of the species-specific ecfX gene target. J Microbiol Methods, 70(1):20–9. 17490767 10.1016/j.mimet.2007.03.008

[10] Wayne P (2018). Clinical and Laboratory Standards Institute: Performance standards for antimicrobial susceptibility testing: Twenty-fourth informational supplement, M100-S28. Clinical and Laboratory Standards Institute (CLSI), 34(1).

[11] Amjad A Mirza I Abbasi S (2011). Modified Hodge test: A simple and effective test for detection of carbapenemase production. Iran J Microbiol, 3(4):189–93. 22530087 PMC3330182

[12] Qu TT Zhang JL Wang J (2009). Evaluation of phenotypic tests for detection of metallo-beta-lactamase-producing *Pseudomonas aeruginosa* strains in China. J Clin Microbiol, 47(4):1136–42. 19213696 10.1128/JCM.01592-08 PMC2668318

[13] Najar Peerayeh S Pirhajati Mahabadi R Pakbaten Toupkanlou S (2014). Diversity of beta-lactamases produced by imipenem resistant, *Pseudomonas aeruginosa* isolates from the bloodstream. Burns, 40(7):1360–4. 24513132 10.1016/j.burns.2014.01.009

[14] Yong D Lee K Yum JH (2002). Imipenem-EDTA disk method for differentiation of metallo-beta-lactamase-producing clinical isolates of Pseudomonas spp. and Acinetobacter spp. J Clin Microbiol, 40(10):3798–801. 12354884 10.1128/JCM.40.10.3798-3801.2002 PMC130862

[15] Yong D Lee Y Jeong SH (2012). Evaluation of double-disk potentiation and disk potentiation tests using dipicolinic acid for detection of metallo-beta-lactamase-producing pseudomonas spp. and Acinetobacter spp. J Clin Microbiol, 50(10):3227–32. 22837321 10.1128/JCM.00818-12 PMC3457450

[16] Flateau C Janvier F Delacour H (2012). Recurrent pyelonephritis due to NDM-1 metallo-beta-lactamase producing *Pseudomonas aeruginosa* in a patient returning from Serbia, France, 2012. Euro Surveill, 17(45):20311. 23153474

[17] Van der Bij AK Pitout JD (2012). The role of international travel in the worldwide spread of multiresistant Enterobacteriaceae. J Antimicrob Chemother, 67(9):2090–100. 22678728 10.1093/jac/dks214

[18] Jovcic B Lepsanovic Z Suljagic V (2011). Emergence of NDM-1 metallo-beta-lactamase in *Pseudomonas aeruginosa* clinical isolates from Serbia. Antimicrob Agents Chemother, 55(8):3929–31. 21646490 10.1128/AAC.00226-11 PMC3147624

[19] Shokri D Rabbani Khorasgani M Fatemi SM (2017). Resistotyping, phenotyping and genotyping of New Delhi metallo-beta-lactamase (NDM) among Gram-negative bacilli from Iranian patients. J Med Microbiol, 66(4):402–11. 28150578 10.1099/jmm.0.000444

[20] Rizek C Fu L Dos Santos LC (2014). Characterization of carbapenem-resistant Pseudomonas aeruginosa clinical isolates, carrying multiple genes coding for this antibiotic resistance. Ann Clin Microbiol Antimicrob, 13:43. 25179208 10.1186/s12941-014-0043-3 PMC4282171

[21] Farzana R Shamsuzzaman S Mamun KZ (2013). Isolation and molecular characterization of New Delhi metallo-beta-lactamase-1 producing superbug in Bangladesh. J Infect Dev Ctries, 7(3):161–8. 23492993 10.3855/jidc.2493

[22] Malkocoglu G Aktas E Bayraktar B (2017). VIM-1, VIM-2, and GES-5 Carbapenemases Among *Pseudomonas aeruginosa* Isolates at a Tertiary Hospital in Istanbul, Turkey. Microb Drug Resist, 23(3):328–34. 27326514 10.1089/mdr.2016.0012

[23] Johnson AP Woodford N (2013). Global spread of antibiotic resistance: the example of New Delhi metallo-β-lactamase (NDM)-mediated carbapenem resistance. J Med Microbiol,62(4):499–513. 23329317 10.1099/jmm.0.052555-0

[24] Mirsalehian A Feizabadi M Nakhjavani FA (2010). Detection of VEB-1, OXA-10 and PER-1 genotypes in extended-spectrum beta-lactamase-producing *Pseudomonas aeruginosa* strains isolated from burn patients. Burns, 36(1):70–4. 19524369 10.1016/j.burns.2009.01.015

[25] Ranjbar R Owlia P Saderi H (2011). Characterization of *Pseudomonas aeruginosa* strains isolated from burned patients hospitalized in a major burn center in Tehran, Iran. Acta Med Iran. 49(10):675–9. 22071644

[26] Sedighi M Vaez H Moghoofeie M Hadifar S Oryan G Faghri J (2015). Molecular detection of metallo-β-lactamase gene blaVIM-1 in imipenem-resistant *Pseudomonas aeruginosa* strains isolated from hospitalized patients in the hospitals of Isfahan. Adv Biomed Res, 4:57. 25802826 10.4103/2277-9175.151872 PMC4361957

[27] Cornaglia G Mazzariol A Lauretti L (2000). Hospital outbreak of carbapenem-resistant *Pseudomonas aeruginosa* producing VIM-1, a novel transferable metallo-beta-lactamase. Clin Infect Dis, 31(5):1119–25. 11073738 10.1086/317448

[28] Yong D Toleman MA Giske CG (2009). Characterization of a new metallo-β-lactamase gene, blaNDM-1, and a novel erythromycin esterase gene carried on a unique genetic structure in *Klebsiella pneumoniae* sequence type 14 from India. Antimicrob Agents Chemother, 53(12):5046–54. 19770275 10.1128/AAC.00774-09 PMC2786356

[29] Kazeminezhad B Rad AB Gharib A Zahedifard S (2017). blaVIM and blaIMP Genes Detection in Isolates of Carbapenem Resistant P. aeruginosa of Hospitalized Patients in Two Hospitals in Iran. Iran J Pathol, 12(4):392–396. 29563936 PMC5844685

[30] Toleman MA Spencer J Jones L (2012). blaNDM-1 is a chimera likely constructed in Acinetobacter baumannii. Antimicrob Agents Chemother, 56(5):2773–6. 22314529 10.1128/AAC.06297-11 PMC3346620

[31] Zavascki AP Barth AL Gonçalves AL (2006). The influence of metallo-β-lactamase production on mortality in nosocomial Pseudomonas aeruginosa infections. J Antimicrob Chemother. 58(2):387–92. 16751638 10.1093/jac/dkl239

[32] Golshani Z Sharifzadeh A (2013). Prevalence of blaOxa10 Type Beta-lactamase Gene in Carbapenemase Producing Pseudomonas aeruginosa Strains Isolated From Patients in Isfahan. Jundishapur J Microbiol, 6(5).

[33] Wei WJ Yang HF Ye Y (2015). New Delhi Metallo-beta-Lactamase-Mediated Carbapenem Resistance: Origin, Diagnosis, Treatment and Public Health Concern. Chin Med J (Engl), 128(14):1969–76. 26168840 10.4103/0366-6999.160566 PMC4717920

[34] Shacheraghi F Shakibaie MR Noveiri H (2010). Molecular identification of ESBL Genes blaGES-blaVEB-blaCTX-M blaOXA-blaOXA-4, blaOXA-10 andblaPER-in *Pseudomonas aeruginosa* strains isolated from burn patients by PCR, RFLP and sequencing techniques. Int J Biol life Sci, 3(6):138–42.

[35] Poirel L Walsh TR Cuvillier V (2011). Multiplex PCR for detection of acquired carbapenemase genes. Diagn Microbiol Infect Dis, 70(1):119–23. 21398074 10.1016/j.diagmicrobio.2010.12.002

[36] Nordmann P Naas T Poirel L (2011). Global spread of carbapenemase-producing Enterobacteriaceae. Emerg Infect Dis, 17(10):1791–8. 22000347 10.3201/eid1710.110655 PMC3310682

